# Oxygen uptake kinetics and speed-time correlates of modified 3-minute all-out shuttle running in soccer players

**DOI:** 10.1371/journal.pone.0201389

**Published:** 2018-08-21

**Authors:** Mark Kramer, Rosa Du Randt, Mark Watson, Robert W. Pettitt

**Affiliations:** 1 Human Movement Science Department, Nelson Mandela University, Port Elizabeth, South Africa; 2 Psychology Department, Nelson Mandela University, Port Elizabeth, South Africa; 3 Rocky Mountain University of Health Professions, Provo, Utah, United States of America; Sao Paulo State University - UNESP, BRAZIL

## Abstract

How parameters derived from oxygen uptake ${\dot{V}O}_{2}$ kinetics relate to critical speed is not fully understood, and how such parameters relate to more sport-specific performances, such as shuttle running, has not been investigated. Therefore, the primary aims of the present student were to examine the ${\dot{V}O}_{2}$ kinetics during all-out linear and shuttle running and compare physiological variables of all-out running to variables measured during a graded exercise test (GXT). Fifteen male soccer players performed a graded exercise test (GXT) and the ${\dot{V}O}_{2}$ kinetics from a series of three different 3-min all-out tests (3MT’s) were evaluated. ${\dot{V}O}_{2}max$ achieved during the GXT did not differ from maximal ${\dot{V}O}_{2}$ achieved during the all-out tests (F = 1.85, p = 0.13) (overall ICC = 0.65; typical error = 2.48 ml∙kg^-1^∙min^-1^; coefficient of variation = 4.8%). A moderate, inverse correlation (r = -0.62, p = 0.02) was observed between *τ* (14.7 ± 1.92 s) and CS (3.96 ± 0.52 m∙s^-1^) despite the narrow SD for *τ*. No differences (p > 0.05) were observed for any of the ${\dot{V}O}_{2}$ kinetics between continuous and shuttle running bouts. The linear running 3MT (r3MT) represents a viable surrogate to the GXT and data beyond CS and D’ may be gleaned by using the bi-exponential speed-time model.

## Introduction

Measurement of the oxygen uptake (${\dot{V}O}_{2}$) responses to constant work exercise performed in various intensity domains is well researched and understood [1–3], yet research where *severe-intensity* exercise is performed using non-constant strategies (e.g. all-out running), has received limited attention [4,5]. Successful performance in athletic activities is dependent on the level of aerobic energy transfer, which in turn is governed by the magnitude and the time course of pulmonary ${\dot{V}O}_{2}$ and muscle *O*_2_ consumption [3]. Measurement of ${\dot{V}O}_{2}$ kinetics can therefore provide valuable insights pertaining to the ventilatory, cardiovascular and neuromuscular responses to a given exercise mode, duration and intensity [2,6,7].

The speed of the increase in the ${\dot{V}O}_{2}$ response, represented by the primary phase time constant *τ* and is reflective of muscle ${\dot{V}O}_{2}$ kinetics [7], towards a steady state (or quasi steady-state) therefore implicates the relative contribution of oxidative and non-oxidative metabolic processes to energy transfer [6]. Greater depletion of high energy phosphates (primarily phosphocreatine [PCr]) and the anaerobic catabolism of glycogen to lactate is experienced when ${\dot{V}O}_{2}$ kinetics are slower and/or the amplitude of the ${\dot{V}O}_{2}$ kinetics are greater [2,6,7]. Faster ${\dot{V}O}_{2}$ kinetic responses (i.e. smaller *τ*) are therefore indicative of healthy and/or fit individuals, whereas slower responses (i.e. larger *τ*) are more representative of unfit or unhealthy individuals [7]. The extent to which the associated ${\dot{V}O}_{2}$ kinetic parameters of male soccer players relate to critical speed is presently not well understood. Furthermore, how such parameters relate to more sport specific performances, such as all-out shuttle running, has not been investigated.

Modern trends in field sports, such as soccer and rugby, have shown increases in playing intensity (i.e. time and distance spent running at speeds exceeding 21 km∙hr^-1^), necessitating a requisite increase in the physical fitness parameters of players [8–10]. High intensity performance is characterized by the ability to sustain a high percentage of maximum oxygen uptake (${\dot{V}O}_{2}max$), with the gas exchange threshold (GET) often being associated with an athlete’s maximum sustainable intensity rate [7,10–12]. However, although the GET is a good predictor of exercise performance, it is not reflective of the athlete’s competition specific intensity [13]. Alternatively, critical power (for cycling) or critical speed (for running) has emerged as a more viable substitute and has been found to be more consistent with high-intensity exercise [7]. Knowing and understanding specific speed thresholds and the physiological responses they elicit therefore has important performance implications.

Exercise intensities performed at speeds below the lactate threshold (LT, or gas exchange threshold [GET]) are defined as moderate, whereby a metabolic steady state is rapidly achieved. Heavy intensity exercise is bounded by intensities above LT, but below critical power (CP or the maximal lactate steady state [MLSS]), resulting in elevated but stable blood lactate levels [2,7]. In fact, CP represents the highest ${\dot{V}O}_{2}$ at which blood lactate and ${\dot{V}O}_{2}$ can be stabilized [2]. From a ${\dot{V}O}_{2}$ kinetics perspective, the ${\dot{V}O}_{2}$ in the heavy intensity domain exhibits a ‘slow component’ which represents an elevated ${\dot{V}O}_{2}$ and results in a delayed steady state of 10–15 minutes or more depending on the relative power/speed within the heavy intensity domain. Exercise in the severe domain is constrained to intensities above CP in which ${\dot{V}O}_{2}max$ can be elicited [14]. Within the severe domain, the slow component causes ${\dot{V}O}_{2}$ to rise to maximum and blood lactate levels to rise exponentially until exercise is terminated [2,10,14].

More specifically, CP has been found to be a robust parameter representative of a fatigue threshold, placed approximately midway between GET and ${\dot{V}O}_{2}max$, which demarcates the heavy from the severe intensity domains [12]. The CP concept was first proposed by Monod and Scherrer whereby maximal work rate and the time to exhaustion of a single muscle group exhibited a hyperbolic relationship [15]. The curvature constant of the hyperbolic relationship (termed W’; measured in kilojoules [kJ]) represents the maximum amount of work that can be completed at intensities above CP. This same relationship has since been extended to whole body exercise such as cycling [16,17], swimming [18,19], rowing [20,21], running [22,23] and even field-based sports such as soccer and rugby [24–26].

What had initially held back the broader implementation of the CP concept was the requirement of several exhaustive bouts over several days [12]. This limitation was overcome in 2006 whereby it was evidenced that a 3-min all-out exercise test (3MT) for cycling was found to accurately replicate the CP and W’ values obtained using the more cumbersome protocols [27]. When running is the preferred mode of exercise, the CP term is replaced with critical speed (CS; measured in m∙s^-1^ as opposed to watts), and W’ is replaced by D’ (measured in meters, and is indicative of the maximum distance that can be covered at speeds above CS). The same 3MT protocol has since been successfully applied to running (here referred to as r3MT to differentiate it from the cycling version) resulting in the successful derivation of CS and D’ [22,23]. The conventional r3MT requires athletes to run all-out in a straight line (or around a track), and has even been used to derive CS and D’ parameters for soccer and rugby players. Sports involving shuttle running, that incorporate multiple changes of direction, may limit the ecological validity of the r3MT, and motivates a modification of the 3MT protocol to incorporate all-out shuttle running. To our knowledge there is presently no research whether modifications of the r3MT protocol would modify the physiological loading of athletes as measured by the ${\dot{V}O}_{2}$ uptake kinetics as well as other physiological measures such as heart rate (HR), breathing frequency (BF), minute ventilation (${\dot{V}}_{E}$), or the respiratory exchange ratio (RER). No studies, to the knowledge of the present authors, have measured the ${\dot{V}O}_{2}$ kinetics during the r3MT or modified versions thereof.

Similarly, given the nature of the r3MT speed-time curve, a wealth of information may be overlooked when only CS and D’ parameters are considered. Factors such as maximal speed achieved, rate of speed decay towards CS, and time to maximal speed are simply not reported in the literature. In part, this is due to a lack of mathematical modeling that accurately represents the instantaneous changes in speed during the r3MT. Such modeling may lead to greater insights into physiological factors governing high intensity running performance.

Given that CS and D’ are mathematically derived parameters, the mathematical model bears important consideration as variation in model selection will influence the parameter estimates [28,29]. Although various mathematical models exist to derive both CP and W’ (or CS and D’), none yet have attempted to model the r3MT. We introduce such a model in the present study and compare the CS and D’ parameters derived to more traditional methods proposed by Vanhatalo et al. [16] and Broxterman et al. [23].

The principal purpose of this study was therefore to characterize the ${\dot{V}O}_{2}$ kinetics of linear all-out running and contrast these to the ${\dot{V}O}_{2}$ kinetics of all-out shuttle running of varying distances (i.e. 25-m and 50-m). We also distinguished the physiological parameters obtained from all-out running to those obtained from a traditional laboratory-based GXT to determine whether the physiological stresses imposed by the all-out tests were inherently different. Given that the speed-time curve of linear all-out running has not been modeled before, but that such modeling could provide important performance-related information that could be used for intervention-based analyses, a secondary purpose of the study was therefore to determine whether such a model could adequately characterize the speed-time curve. The model was compared to traditional methods of analysis for deriving CS and D’, and extended to compare the speed-time model parameters to those of the ${\dot{V}O}_{2}$ kinetics.

## Materials and methods

### Ethics statement

The Research Ethics Committee for human test subjects of the Nelson Mandela University, in accordance with the Code of Ethics of the World Medical Association (Declaration of Helsinki), approved all procedures. All subjects provided written informed consent after having the testing procedures explained both verbally and in written format.

### Experimental overview

Subjects visited the testing facility on five separate occasions, with each visit separated by at least 48 hours over a two-week period. The first visit was used to familiarize subjects with the testing procedures prior to the start of experimentation. On the second visit subjects performed the incremental running test on a motorized treadmill (Woodway, USA) to determine ${\dot{V}O}_{2}max$ and GET, as well as the heart rate (HR), minute ventilation (${\dot{V}}_{E}$), respiratory exchange ratio (RER), breathing frequency (BF) and running velocities associated with these parameters. On the third visit, after a standardized warm-up, subjects completed the r3MT on a 400m outdoor track with a portable spirometer (Metamax 3B, Cortex Biophysik, Leipzig, Germany), global positioning system (GPS, Cortex, Germany) and Polar H7 heart rate monitor (Polar Electro Oy, FI-90440, Kempele, Finland) to determine peak values along with data for subsequent modeling of the ${\dot{V}O}_{2}$ kinetics. The fourth and fifth visits utilized the same assessment set-up as that of the 3MT described previously, but the tests were modified to incorporate all-out shuttle-like turns over 25-m or 50-m distances respectively on a designated portion of the outdoor track to maintain the same surface kinetics. The sequence of the all-out testing was counterbalanced to avoid an order effect.

### Subjects

A total of 15 male soccer players volunteered for the study. The subjects had the following characteristics (mean ± SD): age = 23.1 ± 3.1 years, height = 1.73 ± 0.06 m, and weight = 68.9 ± 8.6 kg. Subjects were recruited from the Nelson Mandela University first team soccer club, were apparently healthy, had a minimum of one-year competitive playing experience, were not taking any medications and were uninjured at the time of testing.

### Procedures

#### Graded exercise test with verification protocol

The system was calibrated prior to each test using ambient air, with an assumed concentration of 20.94% *O*_2_ and 0.03% *CO*_2_, as well as a gas of known *O*_2_ and *CO*_2_ concentrations of 15% and 5% respectively as per manufacturer’s instructions. The turbine flowmeter was calibrated using a 3-L syringe (Metamax 3B, Cortex Biophysik). Prior to the GXT, subjects completed a five-minute warm-up at 6–8 km∙hr^-1^ on a motorized treadmill (Woodway, 4Front, USA), followed by a five-minute rest period during which subjects were encouraged to complete dynamic stretches. The ramp test began at 8 km∙hr^-1^ at an incline of 1^o^ and increased by 1 km∙hr^-1^ every minute until exhaustion was reached. Inspired and expired gas volume and concentrations were continuously sampled breath-by-breath using an automated open circuit spirometry device (Metamax 3B, Cortex Biophysik). Heart rate was continuously monitored throughout the test using short range telemetry (Polar H7 HR monitor, Polar, Finland). A rating of perceived exertion (RPE), using the original Borg scale [30], was used to monitor when athlete’s felt close to exhaustion. Strong verbal encouragement was given throughout the test to ensure maximal effort. Once exhaustion was reached, subject’s straddled the treadmill belt, upon which speed was returned to 6 km∙hr^-1^ to allow for an active recovery period lasting 3-minutes. To determine whether a ‘true’ ${\dot{V}O}_{2}max$ was attained, a verification bout was utilized [31–34]. At the end of the 3-minute active recovery period, the treadmill speed was increased to 2-stages below the speed reached at the final stage of the primary ${\dot{V}O}_{2}max$ test, that is, if the test was initially terminated at 15 km∙hr^-1^ using a 1 km∙hr^-1^∙min^-1^ protocol, then the validation bout would be initiated at 13 km∙hr^-1^ to validate the ${\dot{V}O}_{2}max$ value. The end-stage ${\dot{V}O}_{2}$ reached during the verification bout would need to be within 3% of the original bout to be deemed a ‘true’ ${\dot{V}O}_{2}max$ [33,34].

Gas exchange data were reduced to 10-second averages for the estimation of the GET using the following criteria: (1) the first disparate increase in ${\dot{V}CO}_{2}$ in the ${\dot{V}CO}_{2}$ vs. ${\dot{V}O}_{2}$ plot using the V-slope method [35,36]; (2) an increase in ${\dot{V}}_{E}/{\dot{V}CO}_{2}$ with no increase in ${\dot{V}}_{E}/{\dot{V}O}_{2}$; and (3) the first increase in end-tidal *O*_2_ tension with no fall in end-tidal *CO*_2_ tension. ${\dot{V}O}_{2}max$ was determined using the highest ${\dot{V}O}_{2}$ average over a 30-second period during the GXT, with validation of the ‘true’ ${\dot{V}O}_{2}max$ measured with the verification bout. Oxygen uptake, HR, ${\dot{V}}_{E}$, BF and speed at Δ50% were calculated from the initial GXT data as the midpoint between GET and ${\dot{V}O}_{2}max$ data. The speed at GET (sGET), Δ50% (sΔ50%) and at ${\dot{V}O}_{2}max$ ($s{\dot{V}O}_{2}max$) were linearly interpolated at 1-minute preceding the sample [22]. The verification bout was used to determine whether a ‘true’ ${\dot{V}O}_{2}max$ was reached. Only two subjects failed to be within the 3% cut-off (3.9% and 4.1% respectively) and were subsequently asked to re-do the test within a one-week period. On re-testing, both subjects managed to be within the requisite cut-off and were subsequently retained for analysis.

#### Three-minute all-out running tests

In the third session subjects completed the r3MT on an outdoor 400 m tartan sprinting track with minimal wind conditions and a clear sky. After 10–15 minutes of active warm-ups and dynamic stretching, subjects were fitted with a portable spirometer and global positioning sensor (GPS) sampling at 1 Hz (Metamax 3B, Cortex Biophysik) along with a chest strapped wireless HR monitor (H7, Polar, Finland). The GPS system connects directly to the portable spirometer thereby allowing speed data collection that is congruent with the breath-by-breath data. Subjects were instructed to run all-out with maximal effort throughout the entirety of the test. Although verbal encouragement was provided throughout the test, subjects were neither informed of the elapsed time nor time remaining to discourage pacing. Subjects were instructed to stop once 3 minutes and 5 seconds had elapsed to ensure full GPS coverage. The same procedures, sprinting track, equipment and principles were applied to the modified 3MT’s (25-m and 50-m shuttle 3MT) during the fourth and fifth sessions, each test being separated by at least 48 hours. The modified 3MT’s, unlike the conventional r3MT, incorporated 180^o^ turns over distances of 25-m or 50-m, and were therefore deemed more “sport specific” for activities such as soccer, rugby, and hockey given that the modified tests would require significant accelerations and decelerations for each shuttle. The number of turns required would be inversely proportional to the distance of the shuttle, in other words, 25-m shuttles would require more turns compared to 50m shuttles in the given time. The modified all-out shuttle test has been validated by comparing CS and D’ against several distance time-trials [37].

#### Assessment of oxygen uptake kinetics

For each subject and each 3MT test, breath-by-breath ${\dot{V}O}_{2}$ data were linearly interpolated to give one value per second (averaging increment of 1 s), which were then time aligned to the start of the test.

The oxygen uptake (*O*_2_) kinetics were modeled using a mono-exponential function [2,3,38] expressed as:

$${\dot{V}O}_{2}(t)={\dot{V}O}_{2}(BL)+A_{1}∙(1-e^{-(t-\delta_{1})/\tau_{1}})$$ [1]

where *t* is the time, ${\dot{V}O}_{2}(BL)$ is the average ${\dot{V}O}_{2}$ measured during the final 30 seconds after a 5-minute resting period that followed dynamic warm-ups, *A*_1_ is the asymptotic amplitude above baseline, *δ*_1_ is the time delay of the exponential, and *τ*_1_ is the time constant of the exponential term. To derive the estimates for the parameters, *A*_1_, *δ*_1_ and *τ*_1_, we used unconstrained non-linear regression by least sum of squares (OriginPro 2017 [version 94E], OriginLab, USA).

#### Measuring and modeling speed for the r3MT

**Fig 1:**
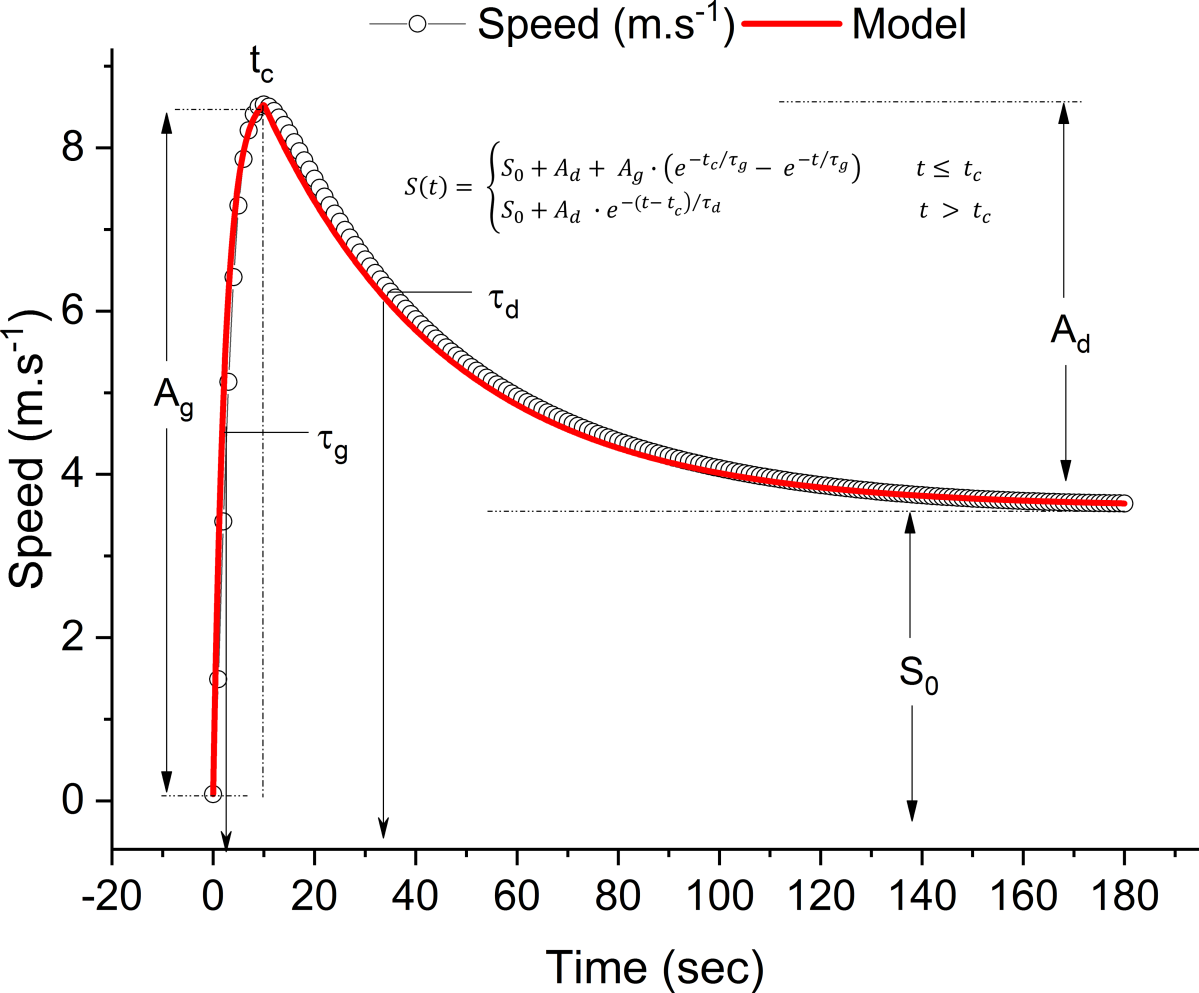
Bi-exponential speed-time (S_0_) model and parameters of the r3MT for a representative subject.

The speed-time data were plotted from the data obtained from the GPS unit. The speed-time curve was modeled using a bi-exponential model similar to that of the oxygen uptake kinetics curve (see Fig 1) [2]:

$$S(t)=\left\{ \begin{array}{l} S_{0}+A_{d}+A_{g}∙(e^{-t_{c}/\tau_{g}}-e^{-t/\tau_{g}})\;t\leq t_{c}\\ {S_{0}+A}_{d}∙e^{-{(t-t}_{c})/\tau_{d}}\;t>t_{c} \end{array}\right.$$ [2]

where *t* is the time, *S*(*t*) is the speed at a given time, *S*_0_ is the y-asymptote (also defined as CS), *A*_*g*_ is the growth amplitude of the exponential, *A*_*d*_ is the decay amplitude of the exponential, *t*_*c*_ is the time offset between exponential growth and decay, *τ*_*g*_ is the time constant of the exponential growth term and *τ*_*d*_ is the time constant of the exponential decay term. Unconstrained non-linear regression by least sum of squares (OriginPro 2017 [version 94E], OriginLab, USA) was used to determine all the coefficients.

From the bi-exponential speed (*S*_0_) model, the CS is represented by the *S*_0_ term, peak speed can be determined by summing the *S*_0_ and *A*_*d*_ terms, and D’ can be determined by integration of the model at speeds above CS (see Fig 1).

Critical speed values were obtained as the average speed of the final 30-seconds for each all-out test [22,37]. Speeds for all tests were sampled at 1Hz, allowing for comparable fidelity of speed-time data for the 25m 3MT and 50m 3MT to that of Saari, et al. [37]. It is important to note however, that greater precision of shuttle speed data should be obtained, and that the values presented should interpreted with some mindfulness.

### Statistical analyses

The Statistica (version 10.1) software package was used for statistical data analysis. Data are presented as mean ± SD unless otherwise stated. All data were assessed for normality using the Shapiro-Wilk test, and all data were found to conform to normality. A one-way analysis of variance (ANOVA) with repeated measures was used to compare maximum ${\dot{V}O}_{2}$ values, ${\dot{V}}_{E},$ *HR*_*max*_ RER, and BF, for each test (GXT, verification bout, r3MT, 25m 3MT and 50m 3MT), followed by a post-hoc Scheffé test for instances where the null-hypotheses were rejected. The parameter estimates for the ${\dot{V}O}_{2}$ kinetics (*A*, *δ*, and *τ*) for all three 3MT’s were analysed using a one-way ANOVA, followed by post-hoc Scheffé testing for significant differences. Simple linear regression was used to compare parameter estimates of the ${\dot{V}O}_{2}$ kinetics and bi-exponential speed-time models. Relative consistency between tests was assessed using the intraclass correlation coefficient (ICC *α*), whereas absolute consistency was evaluated using coefficient of variation (CV%) and typical error (TE) [39]. The fit of the *S*_0_-model to the raw data was evaluated using the coefficient of determination (r^2^) and the standard error of the estimate (SEE). Statistical significance was accepted at a level of p < 0.05.

## Results

### Graded exercise test

Consistent peak ${\dot{V}O}_{2}$ values (i.e. within 3%) in the incremental and verification bouts would provide support that a ‘true’ ${\dot{V}O}_{2}max$ was reached. Relative ${\dot{V}O}_{2}max$ values (ml∙kg^-1^∙min^-1^) between the GXT and the verification bout did not differ significantly (t = 1.73, p = 0.11), and were internally consistent (CV% = 1.7, TE = 0.88 ml∙kg^-1^∙min^-1^), thereby indicating the achievement of a true ${\dot{V}O}_{2}max$. A summary of the physiological parameters obtained from the GXT are presented in Table 1.

**Table 1:** Peak values of the GXT and all-out tests.

	GXT	Verification	GET	Δ50%	r3MT	25m 3MT	50m 3MT	ANOVA Statistics (F, p)
${\dot{V}O}_{2}$ (L∙min^-1^)	3.45 ± 0.29	3.42 ± 0.25	2.65 ± 0.27	3.05 ± 0.26	3.56 ± 0.35	3.71 ± 0.38	3.69 ± 0.33	F = 2.545, p = 0.065
${\dot{V}O}_{2}max$ (ml∙kg^-1^∙min^-1^)	50.46 ± 3.95	49.91 ± 4.05	38.67 ± 3.89	44.57 ± 3.66	51.96 ± 4.56	53.59 ± 4.80	53.03 ± 5.17	F = 1.847, p = 0.130
${\dot{V}}_{E}$ (L∙min^-1^)	126.61 ± 16.92	127.19 ± 18.19	73.63 ± 15.88	100.12 ± 15.22	132.81 ± 15.03	138.34 ± 16.69	137.65 ± 16.90	F = 1.646, p = 0.173
BF (breaths∙min^-1^)	57.87 ± 15.63	59.80 ± 13.43	44.07 ± 15.93	50.97 ± 15.44	60.93 ± 10.72	61.27 ± 8.97	60.60 ± 10.13	F = 0.192, p = 0.942
HR_max_ (beats∙min^-1^)	189 ± 4	189 ± 5	165 ± 8	177 ± 5	183 ± 6^c^*^,^^d^**	179 ± 5^a^*^,^^b^*	182 ± 4^a^**^,^^b^**	F = 10.260, p < 0.001
RER	1.12 ± 0.05	1.02 ± 0.04	0.94 ± 0.03	1.03 ± 0.03	1.25 ± 0.12^a^*^,^^c^**^,^^d^**	1.30 ± 0.08^a^**^,^^b^**	1.30 ± 0.07^a^**^,^^b^**	F = 39.255, p < 0.001
End-stage speed (m∙s^-1^)	4.66 ± 0.36	4.10 ± 0.36	3.14 ± 0.32	3.90 ± 0.31^a^***	3.96 ± 0.52^a^***	3.10 ± 0.36 ^a^***^,^^b^***^,^^d^**	3.66 ± 0.45^a^***	F = 29.928, p < 0.001

Values are mean ± SD. GXT (graded exercise test); GET (gas exchange threshold); 3MT (3-minute all-out run test); 25-m 3MT (3-minute all-out shuttle run test over 25-m distances); 50-m 3MT (3-minute all-out shuttle run test over 50-m distances); ${\dot{V}O}_{2}max$ (maximal rate of pulmonary oxygen uptake); ${\dot{V}}_{E}$ (minute ventilation); BF (breathing frequency); HR (heart rate); RER (respiratory exchange ratio).

^a^ significantly different from GXT

^b^ significantly different from r3MT

^c^ significantly different from 25m 3MT

^d^ significantly different from 50m 3MT

* p < 0.05

** p < 0.01

*** p < 0.001.

Note: verification data was not included in the all-out comparison as this data was used merely to verify the GXT data.

### Comparison in terms of graded and all-out running tests

Table 1 displays the physiological data from the GXT and all-out running performances. No significant differences were found between absolute or relative ${\dot{V}O}_{2}max,\;{\dot{V}}_{E}$, or BF for all physiological tests. Peak values for HR in the all-out tests were lower than the GXT. The RER values were higher during the all-out tests compared to the GXT. From Table 1 above it is evident that CS derived from the r3MT was not significantly different from speed at Δ50% (t = 0.90, p = 0.39) and there was strong internal consistency observed between the two metrics (CV% = 6.8, TE = 0.27 m∙s^-1^).

**Fig 2:**
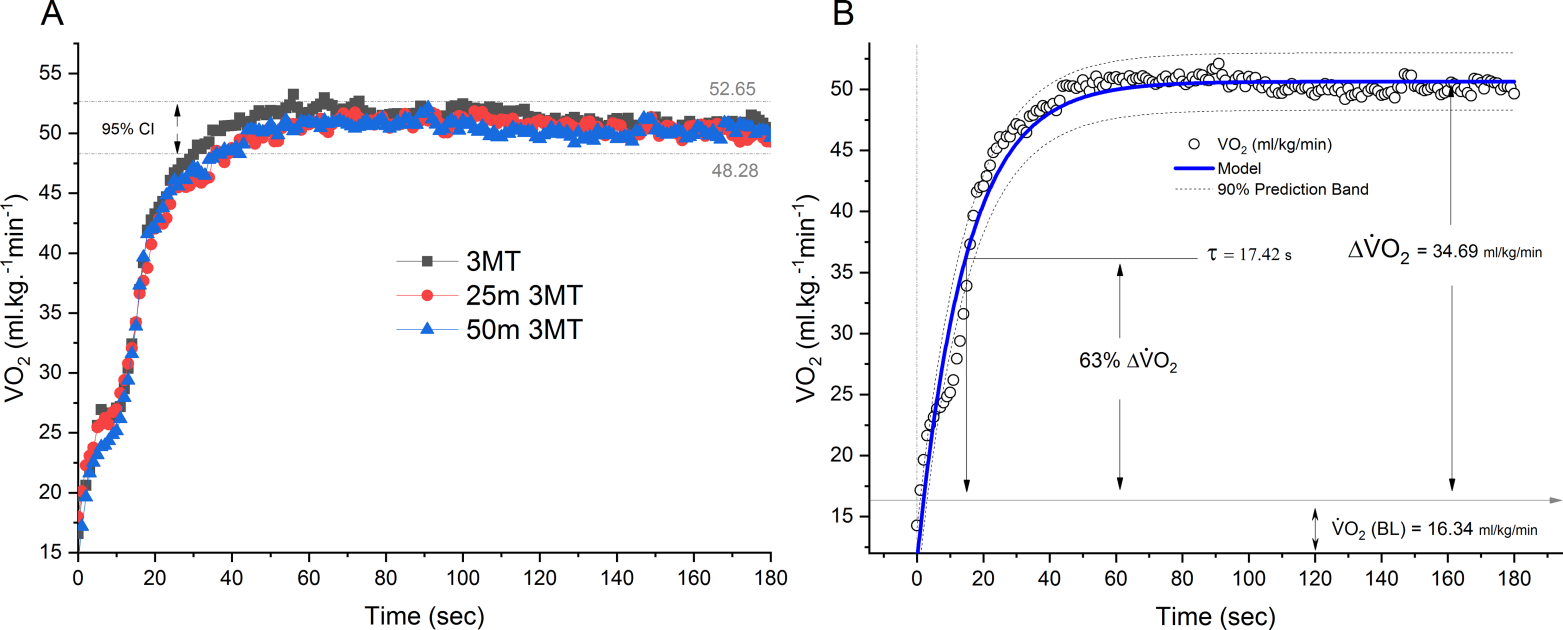
Oxygen uptake for all 3 all-out tests. Panel A: dotted grey lines represent 95% CI of ${\dot{V}O}_{2}max$ derived from the lab-based GXT; Panel B: indicates the summarized parameter estimates of the mono-exponential equation derived for the 25m 3MT for the squad of athletes.

The parameter estimates for the ${\dot{V}O}_{2}$ uptake kinetics for each of the all-out tests are presented in Table 2 (see also Fig 2). No significant differences for any of the parameter estimates could be detected, implying a potentially similar physiological response for each of the all-out tests, at least from a muscle-metabolic and cardiopulmonary perspective. The averaged maximal oxygen uptake obtained for the all-out shuttle tests are presented in Fig 2A together with the 95% confidence interval (CI) for the GXT. An example of the modeled ${\dot{V}O}_{2}$ uptake kinetics for the 25m 3MT is presented in panel B of Fig 2 (R^2^ = 0.97, representing the average goodness of fit for all subjects).

**Table 2:** Parameter estimates for the ${\dot{V}O}_{2}$ response for the all-out tests.

	r3MT	25m 3MT	50m 3MT	ANOVA Statistics (F, df, p)
${\dot{V}O}_{2}(BL)$ (ml∙kg^-1^∙min^-1^)	14.30 ± 5.92	16.34 ± 4.77	12.57 ± 5.63	F = 1.792, p = 0.179
A (ml∙kg^-1^∙min^-1^)	37.39 ± 7.59	34.69 ± 7.01	38.15 ± 8.55	F = 0.827, p = 0.444
Asymptote (ml∙kg^-1^∙min^-1^)	51.69 ± 4.68	51.03 ± 4.70	50.72 ± 5.19	F = 0.154, p = 0.858
*τ* (s)	14.67 ± 1.92	17.42 ± 4.75	14.92 ± 3.38	F = 2.765, p = 0.075
*δ* (s)	0.54 ± 0.31	0.56 ± 0.32	0.47 ± 0.17	F = 0.439, p = 0.647

Values are mean ± SD. ${\dot{V}O}_{2}(BL)$ (baseline ${\dot{V}O}_{2}$); *τ* time constant of the exponential function; *δ* is the time delay of the exponential function.

### The r3MT speed-time (*S*_0_) model

In alignment with the methods described by Vanhatalo et al. [16] and Broxterman et al. [23] for deriving CS from an all-out test (i.e. the average speed of the final 30-seconds of the all-out test), the S_0_ parameter in the present study was compared to the average speed in the final 30-seconds of the r3MT and evaluated for absolute and relative consistency. With a TE of 0.09 m∙s^-1^, CV of 2.3%, and ICC *α* of 0.97, the S_0_ parameter derived from the *S*_0_-model is indeed reflective of CS determined via the methods proposed by Vanhatalo et al. [16] and Broxterman et al. [23]. The same was true for the D’ parameter, which is defined as the area under the curve, but above CS during an all-out test. The D’ parameter would traditionally be determined from the raw speed-time data in the absence of a mathematical model, and may therefore capture ‘noise’ inherent in raw data [16, 23]. As such, a comparison of both methods (i.e. the *S*_0_-model compared to raw data), yielded a TE of 12.31 m, CV of 7.2% and an ICC *α* of 0.93, again indicating strong agreement and consistency. The fit of the modeled speed data to the raw speed data showed a very strong fit (r^2^ = 0.91 and SEE = 0.40 m∙s^-1^). This would imply, at the very least, that the *S*_0_-model is justifiably comparable to the ‘traditional’ methods, and may supersede these methods due to additional information gleaned from the model.

The *S*_0_-model provides a total of 6 parameters (**Table 3**). The CS is evidenced by the S_0_ term, whereas the time at which peak speed (S_max_) is attained is reflected by the t_c_ term. The magnitude of the decay amplitude, which indicates the decline in speed from peak speed to CS, is indicated by the A_d_ parameter, and the decay time constant, reflected by *τ*, represents the amount of time necessary to achieve 63% of A_d_. Finally, an approximation of the peak speed attained is reflected by the *S*_*max*_ parameter, which consists of a summation of the S_0_ and A_d_ terms (see Fig 1).

**Table 3:** Parameter estimates for the r3MT *S*_0_-model.

Parameters	r3MT
*S*_0_ (m∙s^-1^)	3.96 ± 0.52
*t*_*c*_ (s)	7.67 ± 2.54
*A*_*g*_ (m∙s^-1^)	19.13 ± 7.76
*τ*_*g*_ (s)	12.01 ± 8.83
*A*_*d*_ (m∙s^-1^)	5.28 ± 0.78
*τ*_*d*_ (s)	36.95 ± 12.66
*S*_*max*_ (m∙s^-1^)	9.24 ± 0.70

Values are mean ± SD. *S*_0_ (critical speed); *t*_*c*_ (time delay); *A*_*g*_ (growth amplitude); *τ*_*g*_ (growth time constant); *A*_*d*_ (decay amplitude); *τ*_*d*_ (decay time constant); *S*_*max*_ = (*S*_0_ +*A*_*d*_) (peak speed).

**Fig 3:**
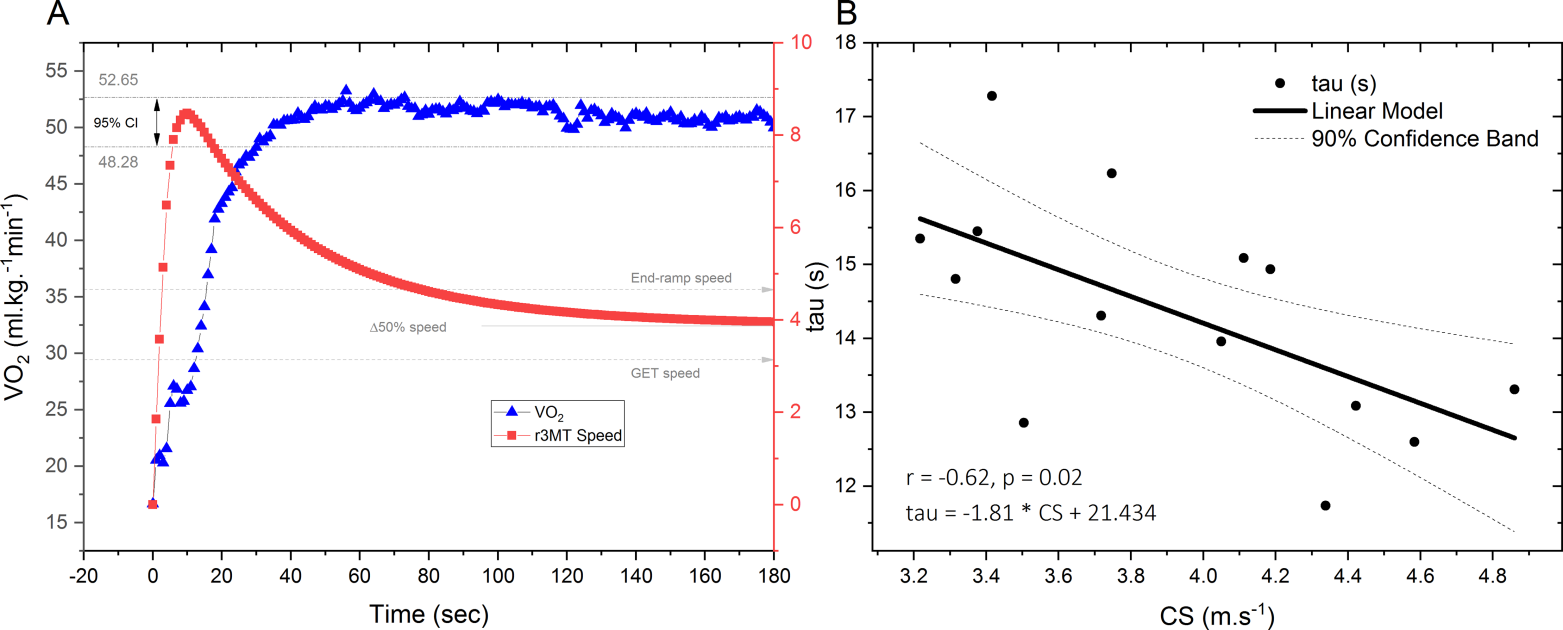
O_2_ kinetics and regression analysis. *(Panel A) O*_*2*_ kinetics and speed-time relationship of the r3MT; (Panel B) regression analysis of the *O*_2_ *kinetics time constant*.

The link between the ${\dot{V}O}_{2}$ uptake kinetics and the *S*_0_-model is presented in Fig 3A. We investigated a potential link between *τ* derived from the ${\dot{V}O}_{2}$ uptake kinetics the CS derived from the r3MT *S*_0_ model. The regression analysis (Fig 3B) yielded a moderate, inverse correlation (Fig 3B).

## Discussion

A surprising finding of the current study was the lack of a significant difference in ${\dot{V}O}_{2}$ kinetics of all three all-out tests. Whether athletes performed a continuous straight-line sprint without directional changes (r3MT), or whether athletes sprinted all-out with 180^o^ turns every 25-m or 50-m respectively, there were no appreciable differences in ${\dot{V}O}_{2}max,\;{\dot{V}}_{E}$, BF, or in parameter estimates of the ${\dot{V}O}_{2}$ kinetic responses such as ${\dot{V}O}_{2}(BL)$, *A*, asymptote, *τ* or *δ* (Tables 1 and 2). Given that ${\dot{V}O}_{2}$ kinetics are reflective of muscle metabolic processes [2,3], this would imply that the physiological, and perhaps neuromuscular, loading of linear and shuttle all-out running are similar. This is an important finding in that, for shuttle running, each change of direction requires substantial braking forces followed by propulsive forces, thereby challenging the force and endurance capacities of the leg musculature [40]. Repeated directional changes would therefore increase the aerobic demand of the legs, with a concomitantly greater level of muscle deoxygenation and fatigue development [40,41]. Conversely, speeds during shuttle running are typically lower compared to straight line running (due to the directional changes), which in turn would lower muscle deoxygenation and fatigue development [40]. The present study therefore lends credence to the latter body of evidence in that ${\dot{V}O}_{2}$ kinetics were not significantly different (eluding to the muscle metabolic processes), and HR_max_ being lower during shuttle running compared to linear running.

Also noteworthy, the ${\dot{V}O}_{2}max$ values achieved in the laboratory were consistently reproduced in all three field-based tests. Attainment of ${\dot{V}O}_{2}max$ during the r3MT is consistent with existing literature [7,12], although much of this has focused almost exclusively on the cycling 3MT. A novel finding therefore lies in the attainment of ${\dot{V}O}_{2}max$ even during the all-out shuttle tests, perhaps hinting at the robustness of the 3MT methodology in taxing the requisite bioenergetic pathways.

Within the all-out bouts, ${\dot{V}O}_{2}max$ was achieved within 90-seconds, specifically ~74 *s* for the r3MT, ~87 *s* for the 25m 3MT, and ~75 *s* for the 50m 3MT, and stayed near constant for the remainder of the tests despite exponential decay in speed that would asymptote in the attainment of CS and a commensurate depletion of D’. These findings are congruent with research by Vanhatalo et al. [42], whereby ${\dot{V}O}_{2}max$ was reached within ~72 *s* during a 3-min all-out cycling test. The attainment, and maintenance, of ${\dot{V}O}_{2}max$ despite a commensurate exponential decrease in running speed is indicative, in part, of a progressive loss of muscle efficiency [42]. This progressive loss has been attributed to a higher phosphate cost of force generation (i.e. mechanistic basis of fatigue) rather than a greater *O*_2_ cost of oxidative of ATP production (i.e. energy supply basis of fatigue] [42].

The rapid attainment of ${\dot{V}O}_{2}max$ during all three 3MT’s can be explained by the fact that speeds above CS lead to substantial decreases in arterial pH, as evidenced by the high RER achieved for all 3MT’s, evoking a dramatic increase in ${\dot{V}}_{E}$ primarily due to increases in BF, hence increasing the *O*_2_ cost of breathing [3,4,7]. The lack of significant differences in ${\dot{V}O}_{2}$ uptake kinetics or physiological correlates, such as ${\dot{V}O}_{2}max,\;{\dot{V}}_{E}$ and BF, between the three all-out tests warrants further investigation (i.e. perhaps investigating the energetics associated with all-out running and/or a muscle-blood profile). From a neurological perspective differences between straight-line running and shuttle running exist [41–44]. All-out sprint exercise requires maximal recruitment of available motor-units thereby requiring increased mitochondrial respiration; hence the increased ${\dot{V}O}_{2}$ uptake towards maximum within 90-seconds of all-out effort [4]. Changes in muscle phosphocreatine (PCr) concentrations, which serve as an indicator of muscle metabolic perturbations, decrease exponentially during the first 30-seconds of all-out activity, after which the rate of utilization tends to asymptote towards a pseudo ‘steady-state’ [3,4]. The muscle metabolic perturbations therefore reach very high levels during such activities, which may in part explain the fatigue experienced during all-out running. Although this may serve as a viable explanation for the exponential speed decrements, other evidence is suggestive of a mechanistic fatigue basis, rather than an energy supply limitation [42].

The CS derived from the *S*_0_-model was comparable to the laboratory-based sΔ50%, a finding consistent with previous investigations using all-out cycling [16] and running [22]. When coupled with the field-based ${\dot{V}O}_{2}max$ data, the implications of the present analyses are that the physiological parameters derived from the r3MT were similar to those obtained from the laboratory-based GXT, meaning that the r3MT may serve as a potential surrogate for the GXT as a measure of aerobic fitness for athletic population groups using portable spirometry.

A potential link between the parameter estimates derived from the ${\dot{V}O}_{2}$-kinetics and the *S*_0_-model was also established, with a moderate, inverse relationship between *τ* and the CS attained during the r3MT (Fig 3). Such a result indicates that individuals with lower time constants had higher overall CS values; in other words, those who exhibited a faster time to ${\dot{V}O}_{2}max$ could sustain a higher overall CS. The *τ* is representative of muscle-metabolic processes, whereby intensity-dependent ${\dot{V}O}_{2}$ closely mirrors the intramuscular [PCr] kinetics in an inverse relationship (i.e. dramatic decreases in [PCr] and other metabolites drive the respiratory response), with the magnitude of the response being dependent on the proximity of the intensity to CS [3,45,46]. The CS parameter attained at the end of an all-out test is reliant on primarily peripheral fatigue related factors such as reduced [PCr], elevated [P_i_] and reduced pH [12,46]. It is important to note however, that at intensities above CS, additional factors may contribute to fatigue (e.g. central fatigue [impaired muscle activation, efficiency]), which could explain some of the exponential speed decay, and may account for some of the unexplained variance between the *τ* and CS parameter comparisons (i.e., the *τ* parameter from the *S*_0_-model may be measuring a unique physiological characteristic) [42]. Future investigators may wish to evaluate experimental interventions (e.g., manipulation of inspired *O*_2_, adaptations to training) on the *τ* parameter from the *S*_0_-model.

The present study presents the first link between a parameter estimate derived from ${\dot{V}O}_{2}$ kinetics and that derived from the *S*_0_-model, tentatively hinting at the utility of the model to provide potentially useful insights to the underlying mechanics of the r3MT (Fig 3B). Future research should therefore focus on probing differences in CS and D’ between the three different versions of the r3MT, as would investigations pertaining to the energetics of turning. In other words, do turning and turning frequencies tend to tax the body to a greater extent compared to straight-line running? It is hypothesized at this stage that the peak speeds attained for each of the three tests would be distinctive, and that the number of turns would be vastly different especially for the 25m 3MT compared to the 50m 3MT [47]. It is inferred therefore that differences in speed and turn quantity may be inversely proportional which may explain the overall similar physiological loads between tests obtained in the present study. It is acknowledged that all-out running, which is non-constant, may limit the utility of spirometry to detect the underlying physiological loads imposed on the human body. Investigating the kinetic energetics of turning may therefore provide insights that differentiate all-out shuttle turning from linear all-out running, whereas at this stage, no discernible differences between the various all-out modalities were apparent.

## Conclusions

The practical findings for the study were four-fold. Firstly, all three 3MT’s yielded ${\dot{V}O}_{2}max$ values similar to laboratory-based assessments implying that the r3MT’s may provide a suitable estimate of ${\dot{V}O}_{2}$ uptake within a three-minute time frame, as well as providing additional parameters such as CS and D’. Secondly, no significant differences in ${\dot{V}O}_{2}$ kinetics could be detected using present methods implying that 25-m or 50-m all-out shuttles could provide a useful alternative for determining ${\dot{V}O}_{2}$ uptake kinetics within the severe-intensity domain. Thirdly, the introduction of a bi-exponential *S*_0_-model may provide useful insights into underlying mechanics of the r3MT. The model may be useful in comparing the different r3MT’s based on the notion that accurate measurements of speed can be made for the all-out shuttle versions of the r3MT. Finally, the ${\dot{V}O}_{2}$ time constant, or *τ*, is inversely related to CS, implying that underlying fatigue mechanisms may be similar; but, further inquiry into the all-out methodology is recommended.

## Abbreviations

${\dot{V}O}_{2}$: rate of pulmonary oxygen uptake
${\dot{V}CO}_{2}$: rate of pulmonary carbon dioxide expulsion
HR_max_: maximum heart rate
BF: breathing frequency
${\dot{V}}_{E}$: minute ventilation
CP: critical power
RER: respiratory exchange ratio
CS: critical speed
GET: gas exchange threshold
Δ50%: midpoint between GET and ${\dot{V}O}_{2}max$
D’: curvature constant of the speed-time relationship for high-intensity exercise (maximum distance covered at speeds above CS)
r3MT: 3-minute maximal run test
TE: typical error
ICC: intra-rater correlation coefficient
CV: coefficient of variation
*O*_2_: oxygen
*CO*_2_: carbon dioxide
